# Mind the Eyes: Artificial Agents’ Eye Movements Modulate Attentional Engagement and Anthropomorphic Attribution

**DOI:** 10.3389/frobt.2021.642796

**Published:** 2021-05-28

**Authors:** Davide Ghiglino, Cesco Willemse, Davide De Tommaso, Agnieszka Wykowska

**Affiliations:** ^1^ Social Cognition in Human-Robot Interaction, Istituto Italiano di Tecnologia, Genova, Italy; ^2^ DIBRIS, Università Degli Studi di Genova, Genova, Italy

**Keywords:** humanoid robot, attentional engagement, intentional stance, mindreading, eye movements

## Abstract

Artificial agents are on their way to interact with us daily. Thus, the design of embodied artificial agents that can easily cooperate with humans is crucial for their deployment in social scenarios. Endowing artificial agents with human-like behavior may boost individuals’ engagement during the interaction. We tested this hypothesis in two screen-based experiments. In the first one, we compared attentional engagement displayed by participants while they observed the same set of behaviors displayed by an avatar of a humanoid robot and a human. In the second experiment, we assessed the individuals’ tendency to attribute anthropomorphic traits towards the same agents displaying the same behaviors. The results of both experiments suggest that individuals need less effort to process and interpret an artificial agent’s behavior when it closely resembles one of a human being. Our results support the idea that including subtle hints of human-likeness in artificial agents’ behaviors would ease the communication between them and the human counterpart during interactive scenarios.

## General Introduction

“Deep,” “sparkling,” “expressive,” “curious,” “sad”: these are only a few of the adjectives that we can use to describe someone’s eyes. Some writers even referred to this sense as the window to the soul, as it can provide information related to others’ mental states, emotions, and intentions (Vaidya et al., 2014). Indeed, every one of us has experienced the feeling to resonate with someone else just at first glance, by making eye contact. If we think about our everyday life, for example, it may happen that we meet a stranger and we immediately understand whether he is sad or happy, just by the look in his eyes (Lee and Anderson, 2017). Neurotypical individuals are usually quite sensitive to the information conveyed by the eyes and are relatively proficient in inferring other agents’ mental states using such a limited source of information (Baron-Cohen et al., 2001). For example, when engaged in a joint action with another person, like moving a heavy object, people are spontaneously inclined to monitor the partner’s eyes to infer his/her mental states (Huang et al., 2015).

The relevance of the ability to “read” mental states through the eyes has been widely studied in the literature. A number of studies demonstrated that understanding another agent’s gaze direction and pattern could be crucial to accomplish a joint task. For example, gaze can cue attention towards an intended object (Sebanz et al., 2006), it can signal interest in an event happening in the environment (Meyer et al., 1998), and even anticipate motor actions (Johansson et al., 2001). Indeed the ability to understand such cues is fundamental in social environments (Butterworth, 1991).

Thousands of years of social interaction contributed to the development of this ability (Hauser, 1996; Scott-Phillips, 2010), to the point that people appear to notice gaze cues even when the agent that is displaying them is artificial (Fiore et al., 2013). This may be due to the spontaneous adoption of cognitive strategies that are similar to those involved in interpreting human-like behaviors displayed by non-human agents (Chaminade et al., 2012). Indeed, behavioral cues that remind of human beings seem to elicit the adoption of such strategies (Abubshait and Wiese, 2017). In particular, past research showed that when artificial agents display behaviors reminiscent of humans, it is likely that individuals ascribe a mind to them (Heider and Simmel, 1944; Abell et al., 2000; Castelli et al., 2000). Also, artificial agents that can express “emotions” at the behavioral level (for example, with facial and bodily expressions) are often rated as more intentional, likable, and human-like than non-expressive ones (for a review, see Hortensius et al., 2018). Even the likelihood that artificial agents are perceived as social partners depends upon their ability to create the “illusion” of possessing intentions and mental states (Wiese et al., 2017).

We speculate that endowing subtle hints of human-likeness in the behaviors displayed by an artificial agent, such as gaze patterns and eye-movements, promotes the implicit association between that agent’s behavior and the behaviors individuals experience during everyday interactions (Banks, 2019). Indeed, even the tendency to attribute a mind towards an artificial agent increases linearly with its perceived human-likeness (Krach et al., 2008). Therefore, equipping artificial agents with a gaze repertoire that is typical of human beings may create the impression that the behavior they display is motivated by mental states and intentions and, consequently, facilitate social attunement^1^ (Wiese et al., 2017). As a cascade effect, this impression would facilitate the understanding of the behaviors that the artificial agent displays and would increase the chance of attributing anthropomorphic traits to the agent. Understanding how these spontaneous associations work would provide useful insights for artificial agents’ developers, and smoothen the inclusion of artificial agents in contexts where the interaction between technology and humans is required (Dautenhahn, 2007).

However, most past research assessed individuals’ perception of anthropomorphism and human-likeness relying mainly on explicit measures (i.e., interviews, questionnaires, self-report scales, etc.) (Loth, 2017). If the final aim is to boost social interaction with artificial agents, qualitative measures collected after the interaction happened might be not suitable to assess the easiness of the interaction. After the interaction, participants report only mental processes that gained access to explicit cognition, while many processes that occur at the implicit level are crucial for the quality of social interaction. Furthermore, judging one’s mental status may follow a qualitative rather than quantitative pattern (i.e., either one ascribes a mind to the agent or does not), which cannot allow for firm conclusions about the mental processes involved while the interaction is happening (Abubshait and Wiese, 2017). Given the importance that gaze has for mindreading (Calder et al., 2002), it would be pivotal to isolate social cognitive mechanisms that are affected by gaze patterns when individuals observe the behavior of artificial agents. Thus, it is fundamental 1) to validate appropriate methods that allow for the evaluation of individuals’ perceptual and attentional processes during the interaction or observation of an artificial agent, and 2) to go beyond explicit attributions of likeability or anthropomorphism (Loth, 2017). Previous research showed, for example, that biologically plausible eye movements displayed by an artificial agent engage an individual’s attention more than mechanistic movements at an implicit, but not explicit level (Ghiglino et al., 2020a). In their study, Ghiglino et al. (2020a), systematically manipulated control parameters of a humanoid robot’s eye movements, to make the robot look more human-like or more mechanistic. By combining participants’ subjective reports with more implicit measures (i.e., eye-tracking metrics), the authors found that the human-like behavior elicited a higher attentional engagement. However, subjective reports were only partially sensitive to the subtle hints of human-likeness displayed by the artificial agent.

Following this line, it is important to explore whether individuals display the same perceptual and attentional mechanisms when processing the behavior of an artificial agent compared to that of a natural agent. By finding ways to analyze systematically attentional and perceptual discrepancies in observing natural and artificial agents, researchers would also be given reliable tools to assess the quality of interactions. Importantly, this approach would grant the possibility to assess mental processes that happen during the interaction, while processing various behaviors of an agent. The present study combines qualitative and quantitative measures, investigating the attentional engagement elicited by natural and artificial agents. Given the aforementioned evidence related to the importance of gaze communication, we decided for the current work to focus solely on eye movements. Thus, to investigate the role of human-like eye movements, we designed two screen-based experiments. Specifically, we explored attentional engagement towards a humanoid and towards a human avatar displaying the same behaviors, which could be either human-like or mechanistic. In the first experiment, we focused on implicit engagement (i.e., attentional focus, decision times) displayed by individuals while observing the behaviors of the two agents. In the second experiment, we explored individuals’ explicit attribution of anthropomorphism towards the robot and the human displaying the same behavior (self-report scales). Finally, we compared the results of both experiments, to understand whether subtle hints of human-likeness affect only implicit processing as well as explicit attribution of anthropomorphic traits.

## Material and Methods—Experiment 1

Our first experiment investigated whether the appearance of the agent (natural vs. artificial), the behavior displayed by the agent (seemingly intentional vs. mechanistic), and the context in which the agent is acting (congruent or incongruent with the behavior) modulate spontaneous attentional engagement, during the observation of other agents involved in a task. As a secondary aim, we explored whether these factors affected the ability to recognize an agent’s behavior during a decision-making task.

### Participants

Fifty-three participants were recruited for this experiment (mean age = 25.2 years *SD* = 5.0, 37 females). All participants reported normal or corrected-to-normal vision and no history of psychiatric or neurological diagnosis, substance abuse, or psychiatric medication. Our experimental protocols followed the ethical standards laid down in the Declaration of Helsinki and were approved by the local Ethics Committee (Comitato Etico Regione Liguria). All participants provided written informed consent to participate in the experiment.

Due to a technical problem with the eye-tracker, we excluded twenty-one participants from data analyses (more than 30% of their data were corrupted). Excluded subjects were all individuals with corrected-to-normal vision wearing glasses or corrective lenses. Despite passing the calibration procedure successfully, a large portion of their eye-tracking data was not recorded. Therefore, our final sample consisted of thirty-two participants (mean age = 24.5 years ±3.63, 22 females).

### Experimental Design

#### Stimuli

To address the aims of our first experiment, we filmed the face of a human actor while he was either actively reading a text on a monitor located in front of him (“intentional,” highly variable behavior in terms of temporal and spatial dynamics) or passively following a dot that was moving across the same monitor (“mechanistic,” repetitive behavior). This latter behavior closely resembled the procedure for calibrating an eye-tracker, requiring the subject to fixate on a dot that appears on the screen in several locations. While the actor was filmed, we recorded his eye movements using a Tobii Pro Spectrum eye-tracker (TobiiAB, Stockholm, 2015). The eye-tracker recorded the Cartesian coordinates of the gaze point relative to the screen during both actions, at a sampling rate of 600 Hz.

Specifically, we collected eye-tracking data of a single participant during two different sessions: 1) gaze following of a calibration marker moving on a screen and 2) gaze reading of a static text on the same screen. Both the sessions were recorded using the same eye-tracking system, display, and screen resolution. Moreover, during the recording, we kept the same distance of the participant with respect to the screen. We stored eye-tracking data in two different output files, one for each session, namely a list of timestamped events containing the 2D Cartesian coordinates of the gaze points of both eyes. Afterward, we implemented an algorithm for replicating the observed behavior by reproducing the human gaze pattern in a humanoid agent, the iCub robot (Metta et al., 2010). Firstly, we programmed a method for transforming the 2D Cartesian coordinates of the human’s eyes into 3D Cartesian coordinates with respect to optical axes of the robot’s eyes. Secondly, since the distance from the screen is a known parameter as well as the screen resolution, we used classical trigonometric methods to extract eye vergence, version, and tilt from the 3D Cartesian in the robot frame. In such a way, we used this triad of values to control the motors of the robot responsible for moving the eyeballs at each timestamp. Finally, we fed the iCub position controller, namely the YARP IPositionDirect (Metta et al., 2006), with the timestamped triad of vergence, version, and tilt to move the robot eyes during the reproduction of the gaze behavior, which was filmed while emulating the human’s behavior. Then, based on the recordings of the human and the iCub, for each agent, we generated two videos where the agents were “Reading a text” and two videos where they were “Calibrating.” The duration of each video was fourteen seconds. The videos of the robot were coupled with the videos of the human so that both agents displayed the very same eye movements (either “Reading” or “Calibrating”) at the same time-frequency^2^.

**FIGURE 1:**
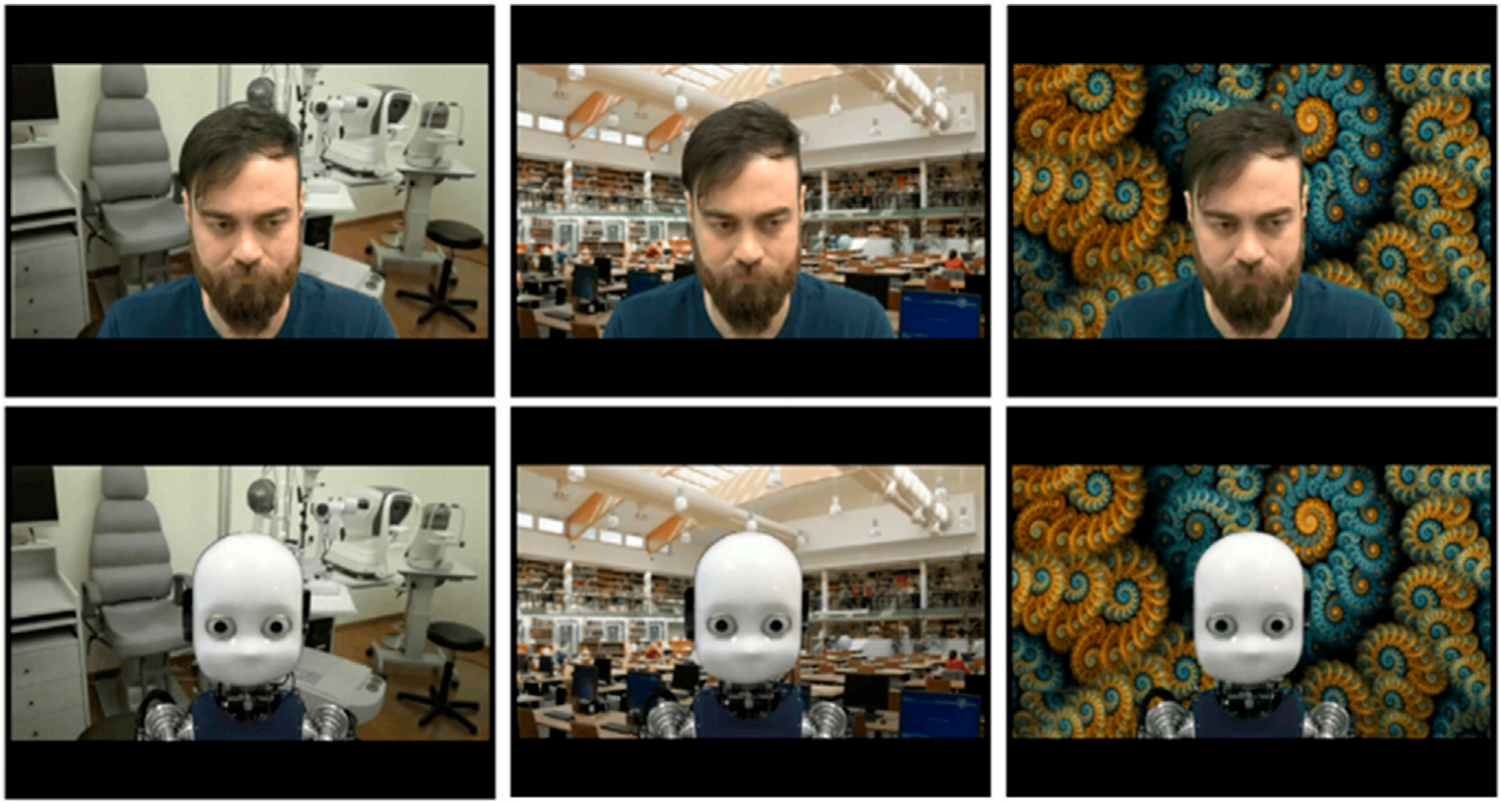
Examples of videos used in the experiment. Other than the behavior of “Calibrating” and “reading” (not displayed), we manipulated the agent and the background. All the stimuli generated for this study were original; the human agent that was filmed and depicted in the image gave explicit consent for the use of the material included in the experiment and in the manuscript.

When the agents were filmed, a uniform green screen was positioned behind them, to cover the full background area. This allowed us to use a basic visual effect technique, where two video streams are composited together. Thus, we superimposed a worldly background over the single-colored backdrop (i.e., the green screen). This allowed us to manipulate the congruency of the background with the agent’s behavior. We defined three types of background scenarios (Context) that we then superimposed on the green screen: 1) library, 2) eye doctor’s study, 3) fractal. We assumed that the “Reading” behavior would be *congruent* with the library scenarios, but *incongruent* with the eye doctor’s study. Besides, we hypothesized that the “Calibrating” behavior could remind participants of the optometry test, therefore being *congruent* with the eye doctor’s study scenarios and *incongruent* with the library. We also added a third scenario, which was abstract and non-informative, as an additional control condition. For each scenario, we selected two pictures (i.e., two libraries, two eye doctors’ studies, two fractals) to add further variability to the stimuli. We generated a pool of 48 stimuli in total^3^ (see Table 1 and Figure 1 for details). Participants saw each video five times across the experiment, which was divided into five blocks interleaved by short breaks. Thus, during each block, each of the 48 videos was displayed once. Thus, we collected performance data and eye-tracking data during 240 trials per subject.

**TABLE 1:** Pool of stimuli generated for the current experiment. Numbers in brackets refer to the two different versions of the behaviors and contexts we generated for the current experiment.

Agent	Behavior	Context
Human	Reading (1)	Library (1)
Human	Reading (1)	Eye Doctor (1)
Human	Reading (1)	Fractal (1)
Human	Reading (2)	Library (1)
Human	Reading (2)	Eye Doctor (1)
Human	Reading (2)	Fractal (1)
Human	Reading (1)	Library (2)
Human	Reading (1)	Eye Doctor (2)
Human	Reading (1)	Fractal (2)
Human	Reading (2)	Library (2)
Human	Reading (2)	Eye Doctor (2)
Human	Reading (2)	Fractal (2)
Human	Calibrating (1)	Library (1)
Human	Calibrating (1)	Eye Doctor (1)
Human	Calibrating (1)	Fractal (1)
Human	Calibrating (2)	Library (1)
Human	Calibrating (2)	Eye Doctor (1)
Human	Calibrating (2)	Fractal (1)
Human	Calibrating (1)	Library (2)
Human	Calibrating (1)	Eye Doctor (2)
Human	Calibrating (1)	Fractal (2)
Human	Calibrating (2)	Library (2)
Human	Calibrating (2)	Eye Doctor (2)
Human	Calibrating (2)	Fractal (2)
Robot	Reading (1)	Library (1)
Robot	Reading (1)	Eye Doctor (1)
Robot	Reading (1)	Fractal (1)
Robot	Reading (2)	Library (1)
Robot	Reading (2)	Eye Doctor (1)
Robot	Reading (2)	Fractal (1)
Robot	Reading (1)	Library (2)
Robot	Reading (1)	Eye Doctor (2)
Robot	Reading (1)	Fractal (2)
Robot	Reading (2)	Library (2)
Robot	Reading (2)	Eye Doctor (2)
Robot	Reading (2)	Fractal (2)
Robot	Calibrating (1)	Library (1)
Robot	Calibrating (1)	Eye Doctor (1)
Robot	Calibrating (1)	Fractal (1)
Robot	Calibrating (2)	Library (1)
Robot	Calibrating (2)	Eye Doctor (1)
Robot	Calibrating (2)	Fractal (1)
Robot	Calibrating (1)	Library (2)
Robot	Calibrating (1)	Eye Doctor (2)
Robot	Calibrating (1)	Fractal (2)
Robot	Calibrating (2)	Library (2)
Robot	Calibrating (2)	Eye Doctor (2)
Robot	Calibrating (2)	Fractal (2)

Given the exploratory nature of this study, we opted for a screen-based experiment instead of a real-time interaction, mainly considering the potential confounding effect due to the non-predictable variability of human behavior. Indeed, variability is a key component of human behavior (Dodge, 1931), to the extent that even the same movement repeated over and over again might assume different kinematic patterns (Stergiou and Decker, 2011). An embodied version of the experiment would not have allowed us to have control over such variability.

#### Procedure and Apparatus

Before starting the experiment, we informed participants about the content of the videos we generated, showing example videos of both agents displaying the “Reading” and the “Calibrating” behaviors. During this familiarization phase, we informed them that the two displayed behaviors corresponded either to the “Reading” or to the “Calibrating.”

During the experiment, videos were presented on a 23.8′′ LCD screen (resolution: 1920 × 1,080). Participants’ head position was limited by a chinrest that was mounted at the edge of the table, at a horizontal distance of 60 cm from the screen. We recorded the participants’ binocular gaze data with a screen-mounted Tobii Pro Spectrum eye-tracker with a sampling rate of 600 Hz. The illumination of the room was kept constant throughout the experimental sessions. Videos and questions were displayed with OpenSesame 3.2.8 (Mathôt et al., 2011).

We instructed participants to carefully watch the videos to detect, as quickly as possible, whether the behavior displayed by the agent was either “Reading” or “Calibrating.” Participants provided their responses by pressing the buttons of a keyboard corresponding to the letters M and Z, counterbalanced across the blocks. After providing their response, participants were asked to rate the confidence in their decision. When this last rating was provided, or in case of a timeout, a new trial started, with a fixation cross presented for five seconds.

Participants’ decision times (DTs), the accuracy of the detections, and confidence ratings were saved along with the eye-tracker data (fixation duration and fixation count).

All participants were debriefed upon completion of the experiment, to explain the purposes of the study to them and to gather qualitative data that could improve the future follow-up study (i.e., their opinions regarding the duration of the experiment, the quality of the video, the nature of the behaviors, etc.).

### Analyses

To explore the effects of Agent, Behavior, and Context on the participants’ attentional engagement during the task, we adopted various mixed models on our eye-tracking data, using RStudio Team, (2015). We defined three main areas of interest (AOI) a priori: 1) the area corresponding to the eye region of the agents; 2) the area corresponding to the face region of the agents (excluding the eyes); and 3) the area corresponding to the background behind the agents (excluding the face). 79.07% of total fixations were recorded within the first AOI (eye region), 5.84% within the second (face region), and 15.09% within the third (background region). Considering the insufficient amount of data points in the non-eye AOIs, we focused our analyses mainly on the eye region. We excluded trials in which the participants provided the incorrect attribution (less than 1% of the total trials) from the analysis.

Fixation duration was the dependent variable of a linear mixed model. Agent, behavior, and context were treated as fixed factors and the subjects’ intercept as a random factor. Then, we converted each participants’ fixation count relative to each AOI into fixation proportions (i.e., the ratio of fixations directed towards each AOI compared to the total number of fixations). Considering the negatively skewed distribution of fixation proportion on the eye region, data were arcsine transformed before the analyses. Then, the arcsine transformed fixation proportion on the eye region was included as the dependent variable of another mixed model, where agent, behavior, and context were treated as fixed factors and the subjects’ intercept as a random factor.

Finally, we analyzed participants’ DTs with an additional linear model. We adopted a minimal a priori data trimming (Harald Baayen and Milin, 2010). Given the positively skewed distribution of DTs, we applied a logarithmic transformation to the data. Then, log-transformed DTs were included as the dependent variable of a final mixed model, where agent, behavior, and context were treated as fixed factors and the subjects’ intercept as a random factor.

To compensate for the lack of consensus on the calculation of standardized effect sizes for individual model terms (Rights and Sterba, 2019), for each model we calculated parameters estimated (*β*) and their associated *t*-tests (*t*, *p*-value) using the Satterthwaite approximation method for degrees of freedom. Furthermore, for each parameter estimated we reported the corresponding bootstrapped 95% confidence intervals. We reported mean values of each dependent variable divided by conditions in the Supplementary Materials to ease the reading of the results. To avoid redundancy, in the main text we reported only statistics relative to significant results. Non-significant results can be found in the Supplementary Materials, along with the original script used for data analysis. For each dependent variable, we explored: 1) Three-way interactions (i.e., the effects due to the interplay between Agent, Behavior, and Context all together); 2) Two-way interactions (i.e., the effects due to the interplay between factor dyads; that are Agent and Behavior, Agent and Context, and Behavior and Context); 3) Main effects (i.e., the effects due to the factors alone, without considering the interactions between them).

## Results—Experiment 1

### Fixation Duration

**FIGURE 2:**
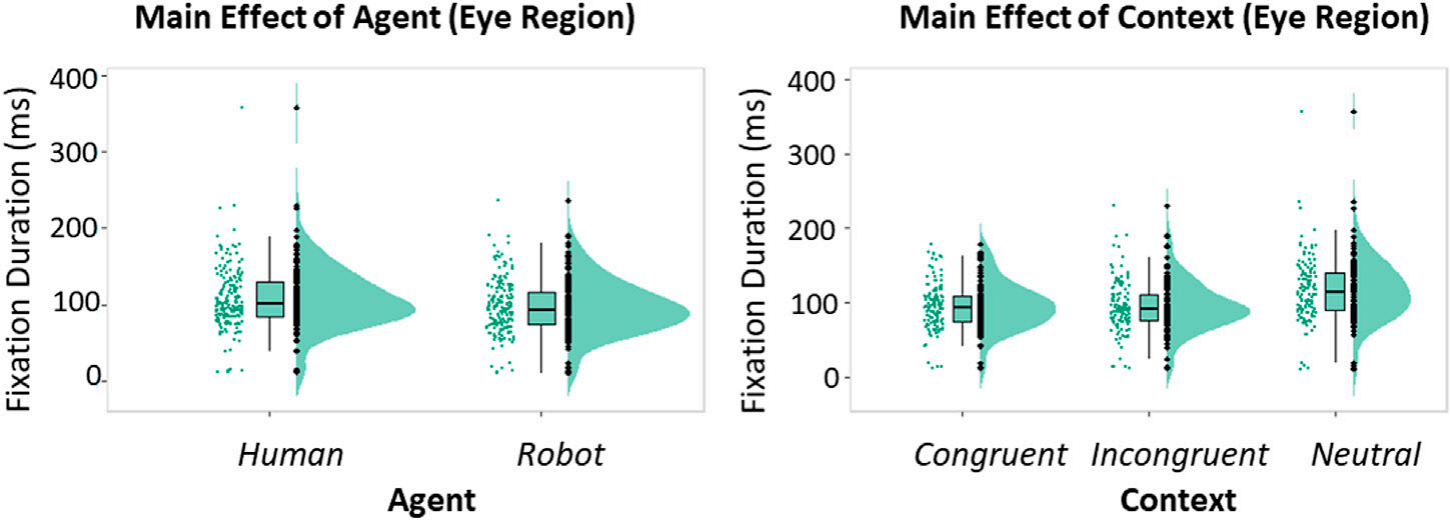
Raincloud plots showing the fixed effect on Fixation Duration due to the main effects of Agent **(A)**, and Context **(B)** (GLM). Boxplots associated with the raincloud plots depict median values (black horizontal lines), interquartile ranges (black boxes), and upper-lower quartile intervals (black whiskers).

To assess the effect of the Agent, its Behavior and the surrounding Context on attentional processing, we first analyzed the inter-trial differences in fixation duration. No effect due to the interaction between Agent, Behavior and Context on fixation duration was found (all *p*-values > 0.05). However, looking at the single factors, we found a main effect of Agent [*β* = −11.80, *t* (340) = −11.80, *p* = 0.028, 95% *CI* = (−22.14, −1.45); Figure 2A] and a main effect of Context [*β* = 19.01, *t* (340) = 3.52, *p* < 0.001, 95% *CI* = (8.57, 29.45); Figure 2B]. Planned comparisons revealed that longer fixations occurred when the Agent was Human compared with the Robot (*t* (340) = 4.73, *p* < 0.001), and when the Context was Non-Informative compared with both Congruent and Incongruent contexts (Congruent vs Non-Informative: *t* (340) = −7.74 *p* < 0.001; Incongruent vs. Non-Informative: *t* (340) = −7.83, *p* < 0.001).

### Fixation Proportion

**FIGURE 3:**
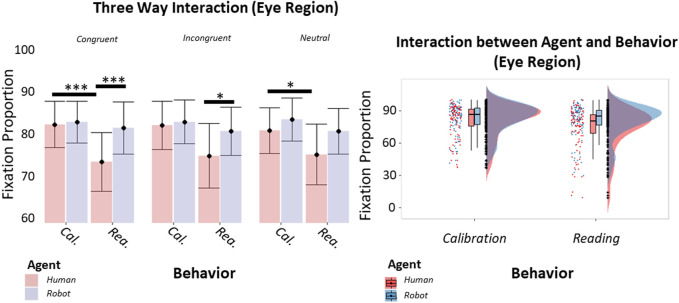
Histograms and raincloud plots showing respectively the thee-way interaction between Agent, Behavior, and Context **(A)** and the two-way interaction between Agent and Behavior **(B)** (GLM). Vertical bars of the histograms denote ±1 standard error, dots denote mean values, horizontal bars denote differences surviving post hoc comparison. Asterisks define the level of significance of the comparison (*p < 0.05, **p < 0.01, *p* < 0.001). Boxplots associated with the raincloud plots depict median values (black horizontal lines), interquartile ranges (black boxes), and upper-lower quartile intervals (black whiskers).

We also analyzed the effects of the Agent, its Behavior and the Context on fixation proportion. Here, our analysis indicated a significant three-way interaction between Agent, Behavior and Context [*β* = −0.08, *t* (341) = −2.01, *p* = 0.045, 95% *CI* = (−0.16, −0.01); Figure 3A] and a significant two-way interaction between Agent and Behavior [*β* = 0.10, *t* (341) = 3.65, *p* < 0.001, 95% *CI* = (0.05, 0.16); Figure 3B]. Planned comparisons showed that participants tended to fixate more often on the eye region of the human during the Calibrating behavior rather than during the Reading behavior, when the context was congruent (*t* (341) = 5.85, *p* < 0.001). A similar difference between the behaviors was found when the context was non-informative (*t* (341) = 3.33, *p* = 0.045). Similarly, when the context was congruent, participants tended to fixate more often on the robot than on the human when these agents were displaying the Reading behavior (*t* (341) = 5.42, *p* < 0.001). Likewise, we found a difference between the agents when the context was incongruent (*t* (341) = −3.58, *p* = 0.020). These results were confirmed by planned comparisons performed on the two-way interaction, highlighting that the behavior that required fewer fixations was the human’s Reading compared to the human’s Calibrating (*t* (341) = −7.86, *p* < 0.001) and to the robot’s Reading (*t* (341) = −7.01, *p* < 0.001). Overall, the Reading behavior required a lower amount of fixations than the Calibrating behavior, as highlighted by the main effect of the Behavior [*β* = −0.12, *t* (341) = −5.85, *p* < 0.001, 95% *CI* = (−0.16, −0.08)] and subsequent planned comparisons (*t* (340) = −7.30, *p* < 0.001). The interaction was paralleled by a main effect of the Agent [*β* = −0.18, *t* (32, 341) = −6.32, *p* < 0.001, 95% *CI* = (−0.24, −0.13)], indicating that participants were faster to identify the behavior when displayed by the robot (*t* (341) = 7.87, *p* < 0.001). Finally, we found a main effect of the Behavior too [*β* = −0.22, *t* (32, 341) = −7.71, *p* < 0.001, 95% *CI* = (−0.28, −0.17)], indicating that the Reading behavior was faster to identify than the Calibrating behavior (*t* (341) = 12.34, *p* < 0.001).

### Decision Times

**FIGURE 4:**
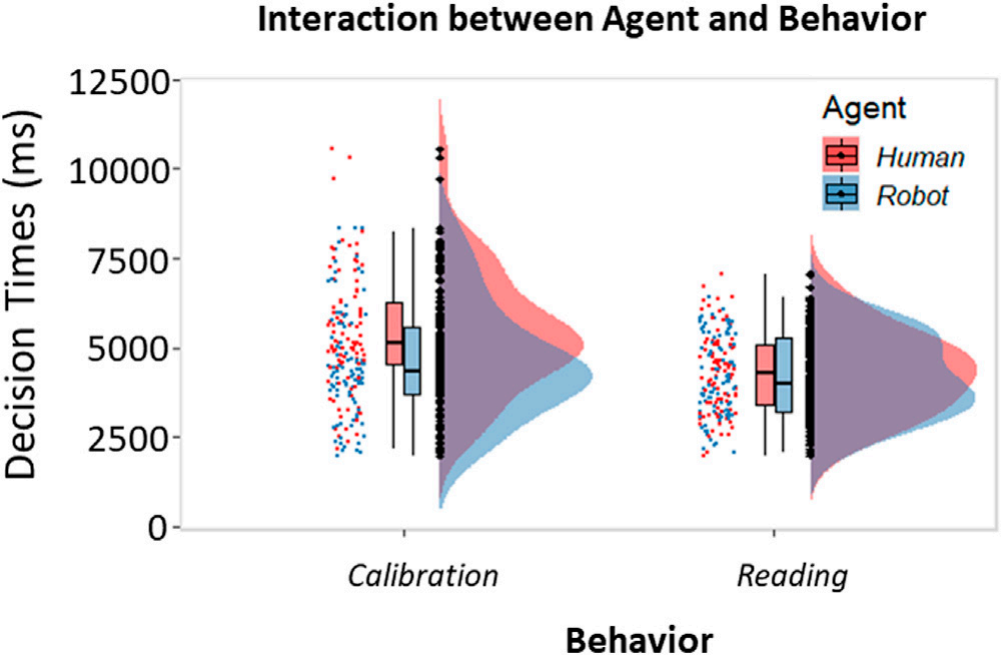
Histograms and raincloud plots showing the two-way interaction between Agent and Behavior (GLM). Boxplots associated with the raincloud plots depict median values (black horizontal lines), interquartile ranges (black boxes), and upper-lower quartile intervals (black whiskers).

To investigate the effect of Agent, Behavior, and Context on the ability to recognize behaviors during the task, we also analyzed our participants’ decision times. The analysis pointed out a two-way interaction between the Agent and the Behavior [*β* = 0.17, *t* (341) = 4.25, *p* < 0.001, 95% *CI* = (0.09, 0.25); Figure 4]. Planned comparisons revealed that the behavior that took longer to identify was the Calibrating behavior displayed by the human when compared to the robot Calibrating (*t* (341) = 10.19, *p* < 0.001) or to the human Reading (*t* (341) = 13.36, *p* < 0.001). Furthermore, the Calibrating behavior displayed by the robot took longer to be identified than the Reading behavior displayed by the same agent (*t* (341) = 4.10, *p* < 0.001).

## Discussion—Experiment 1

With this experiment, we investigated attentional engagement during a novel task that required the observation of a human and a robot displaying the same set of behaviors. The results indicated that participants displayed longer fixations towards the eye region of the human compared with the same region of the robot. Fixation duration is often used as an implicit measure of attentional engagement (Nummenmaa et al., 2006; Ghiglino et al., 2020a). Longer fixations are thought to indicate higher interest than shorter ones (Geisen and Bergstrom, 2017). Indeed, a human agent might engage individuals’ spontaneous attention more than an artificial agent, due to the natural acquaintance people have with their conspecifics (Byrne, 1991).

The interaction effects we found on fixation proportion are in line with this hypothesis. We found a lower fixation proportion on the eye region of a human agent who was reading, relative to the other conditions, which shows that participants distributed their fixation rather on other areas, such as the face and background regions. In other words, participants distributed their fixations on the area surrounding the eyes mainly when the agent was the human, and when he was displaying the reading behavior. Conversely, individuals explored the face and the background regions less when the agent was the robot relative to the human, and when the behavior was calibrating compared with reading. This suggests that, during the task, participants’ attentional resources were focused almost solely on the eye movements of the agent when the agent was artificial, and when the behavior was “mechanistic.” The ratio of on-target vs. all-targets fixations (i.e., the proportion of fixations on a specific area) is often associated with the processing of critical visual information (Holmqvist et al., 2011). We, therefore, conclude that participants required less attentional efforts to interpret the behavior that they were able to relate to the most (i.e., the reading), especially when the human face, to whom we are more accustomed to, displayed it. Indeed, understanding intentional behaviors should be easier than attempting to identify mechanistic ones (Mele and William, 1992).

This is in line with the results we found on participants’ decision times. Specifically, we found that reading behavior was relatively fast to identify, while the calibrating behavior required more time to be recognized. Importantly for the aim of the study, the condition that costs the longest decision time corresponded to the stimuli where the human was displaying the calibrating behavior as if observing an “intentional” agent that displays a mechanistic behavior requires higher processing effort. Interestingly, participants were faster in recognizing both behaviors when the robot displayed them than when the human was. This peculiar effect can be explained by taking into account the expectations that individuals might have towards the two agents. From a purely anecdotal point of view, during the debriefing, a small group of participants reported that they were surprised seeing the human behaving “like a robot” (i.e., during the calibrating behavior). We claim that humans approach artificial agents and their conspecifics with different attitudes that could modulate the way they interpret behaviors (Hinz et al., 2019). Based on our results, we can also speculate that participants were expecting the robot to display a variety of behaviors (i.e., to behave like a human), but they were not expecting the human to behave in a repetitive, mechanistic way (i.e., to behave like a robot).

Along with the effects of Agent and Behavior, we also found the effect of Context on attentional processing. In particular, when the Context was non-informative, participants’ fixations on the eye region were longer. This may indicate that our participants were more engaged by both agents’ behaviors when they were not distracted by the semantic content of the context (i.e., when the context was congruent and incongruent). This is in line with past research investigating the relation between local con global features of visual information (De Cesarei and Loftus, 2011). Indeed, the presence of a “realistic” context might have distracted our participants from the behavior and the agent, attracting their attention towards the background. Thus, the cognitive cost associated with the processing of Congruent and Incongruent backgrounds could explain the presence of shorter fixation on the eye region of both agents. The three-way interaction we found on fixation proportions is in line with this hypothesis; indicating that the interaction between the Agent and the Behavior is particularly strong when the Context is congruent with the Behavior. Thus, we can claim that context could prime the attention towards local cues.

Taken together, these findings highlight the complex interplay between visual information and attentional engagement, suggesting that intentional agents and seemingly intentional behaviors spontaneously attract individuals’ attention. However, it might also be the case that the effects we discussed could be biased by familiarity. Perhaps both the Human-agent and the reading behavior were simply more familiar to the participants than the Robot who was calibrating, respectively. Indeed, we had to provide examples of the calibrating behavior to participants before the experiment, as it is not common behavior for a human being. In a natural environment, this kind of behavior is displayed only during medical visits (eye-exam). On the contrary, reading is an action commonly used in everyday life, and this might have facilitated individuals in the early detection of such behavior. Therefore, the results we found with Experiment 1 might have been biased due to the disparity of the behaviors we selected in terms of prior exposure.

Therefore, after Experiment 1, we needed to clarify whether the effects we found could be explained with reference to the familiarity participants had with the two behaviors, rather than with reference to the degree of intentionality displayed in the behaviors. For this reason, we designed a second experiment, in which we focused more on the self-report impressions that a second group of participants had towards the behaviors used in Experiment 1. Thus, we tested the familiarity of the participants with the behaviors along with their attribution of anthropomorphic traits towards the human and the robot.

## Material and Methods—Experiment 2

Our second experiment investigated how individuals explicitly interpret the behaviors displayed by two different agents, namely the iCub robot and a human. We exposed our participants to a number of videos depicting the humanoid and the human engaged in certain activities on a computer, and we asked them to infer what the agent was doing. We explored our participants’ spontaneous attributions as well as their tendency to attribute anthropomorphic traits towards the two agents. This allowed us for a deeper comprehension of the results we found in Experiment 1.

### Participants

Fifty participants took part in this experiment and were tested *via* Prolific (Prolific, Oxford, UK, 2015), an online recruiting platform (mean age = 26.1 ± 6.0, 20 females). All participants reported normal or corrected-to-normal vision and no history of psychiatric or neurological diagnosis, substance abuse, or psychiatric medication. All participants declared that their first language was English. Each participant provided a simplified informed consent (adapted for online studies) before the beginning of the experiment. All participants that took part in this follow-up experiment were naïve to the videos, and they did not participate in Experiment 1. Our experimental protocols followed the ethical standards laid down in the Declaration of Helsinki and were approved by the local Ethics Committee (Comitato Etico Regione Liguria).

### Stimuli and Apparatus

To address the aim of our second experiment, we used the same pool of stimuli used in Experiment 1, with a few modifications. Since here we were interested exclusively in the interpretation of the behavior as a function of the agent displaying it, we removed the background information from the videos (i.e., we used the original green-screen background). We also included a third behavior that we filmed at the same time as the calibrating and the reading behaviors, which corresponded to the agents Watching movies. We excluded this behavior from Experiment 1, as we wanted to have a clear distinction between the active, more “mentalistic” behavior (i.e., reading) and the passive, more “mechanistic” behavior (i.e., calibrating). Human eye movement while watching movies is a visually-guided behavior, but it is not purely stimulus-driven and might constitute a fuzzy category between “intentional” and “non-intentional” behaviors (Peters and Itti, 2007). Furthermore, in Experiment 2 we also wanted to clarify whether the differences between the calibrating and reading (found in Experiment 1) were due to the familiarity with the behaviors (i.e., calibrating being unfamiliar to most of the participants) or to the proprieties of the behavior (i.e., mentalistic vs. mechanistic). Thus, by adding Watching, we included an additional behavior that was qualitatively different from the reading yet with similar familiarity. Consequently, we extracted a pool of 12 videos fitting a two by three repeated-measures design^4^. Given the more qualitative approach, each video was repeated only twice across experiment two, mainly to check the coherence of participants’ responses.

### Procedure

We ran the experiment online, using Prolific to recruit participants and SoSci Survey (Leiner, 2016) to present the stimuli and collect individuals’ responses. We instructed participants to carefully watch the videos depicting the human and the iCub robot engaged in multiple activities on a computer screen. Before the beginning of the experiment, we asked participants to think about all the activities that a person can do with a computer (i.e., playing videogames, browsing, taking part in a meeting, etc.) and that their task would be to infer what the agents depicted in the videos were doing when we filmed them. Participants were allowed to type their answers without a word limit. After providing their attributions, participants were asked to report whether the behavior displayed by the agent looked familiar to them (two-alternative forced-choice: yes/no), and to rate, on a 10-point Likert scale, how much the agent was aware, focused, and interested, as well as the naturalness of the displayed behavior.

### Analyses

We extracted the verbs used by the participants to describe the actions depicted in the videos. Then, we converted each verb into its non-personal form (gerund). Thus, for each video, we excerpted fifty verbs describing the behavior enacted by the agent, according to participants’ answers. We then performed a text mining analysis on the verbs to determine the frequency of their use across the entire experiment. Then, we compared the frequencies of the most common verbs across conditions, using a series of generalized linear mixed models (GLMM) in R Studio. Agent and Behavior were treated as fixed factors of the model, and the subjects’ intercept was treated as a random factor. Given the nature of our dependent variable (frequency of use), Poisson’s frequency distribution was used as a reference function for the models.

Separately, we analyzed the familiarity reported by participants with each video. We used a GLMM to compare conditions. Agent and Behavior were treated as fixed factors and the subjects’ intercept was treated as a random factor. Since the dependent variable was binary (familiarity), we used the binomial distribution as the reference function of the model.

Finally, we analyzed participants’ ratings on their perceived naturalness of the behavior as well as their ratings on perceived awareness, focus, and interest displayed by the agent. Considering the negatively skewed distribution of ratings, data were arcsine transformed before the analyses. Then, we applied a series of linear mixed models (GLM) to investigate the effects of the Agent and Behavior, treated as fixed factors, on the ratings, given the subjects’ intercept as a random factor.

To clarify whether the effects found on the ratings could be better explained by participants’ familiarity with the behaviors, rather than by our experimental design, we estimated four final alternative linear models that comprised familiarity as the only fixed factor and each rating as a dependent variable. Then, we evaluated the adequacy of each model fit based on a Chi-square difference test and the Akaike’s Information Criterion (AIC) associated with each model.

## Results—Experiment 2

**FIGURE 5:**
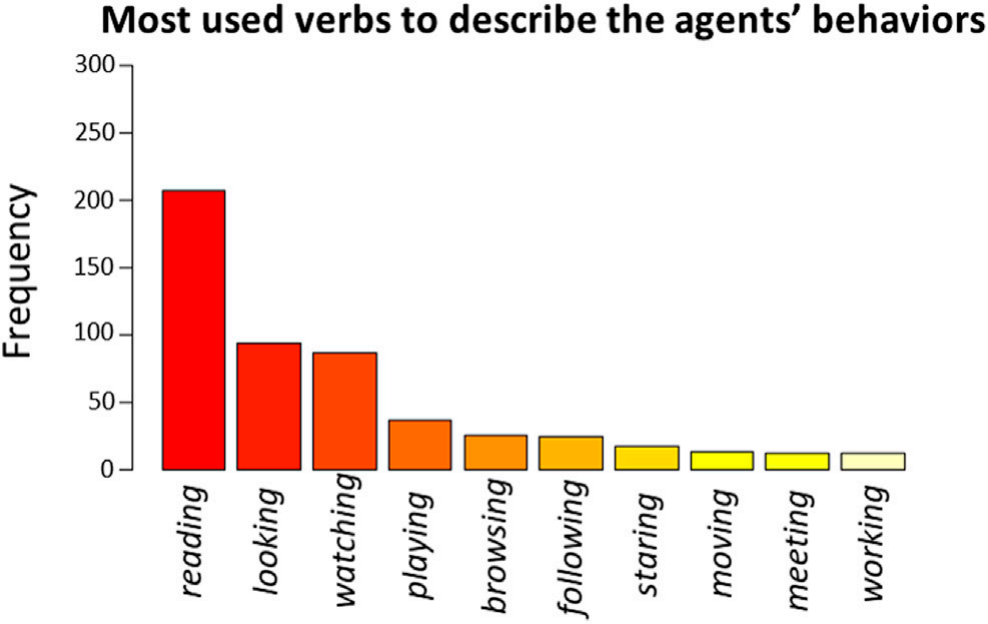
Frequency plot of the ten most used verbs used by participants to describe the agents’ behaviors.

The ten most used verbs to describe the agents’ behaviors were: reading (count = 207), looking (count = 94), watching (count = 86), playing (count = 36), browsing (count = 25), following (count = 24), staring (count = 17), moving (count = 13), meeting (count = 12), working (count = 12) (Figure 5). Only the first three verbs led to converging models, therefore we excluded all the other verbs from data analysis to avoid overfitting of data (Finnoff et al., 1993). Regarding the frequency of use of the verb “reading,” we found a significant main effect of the Behavior [*β* = 2.69, *t* (293) = 6.37, *p* < 0.001, 95% *CI* = (1.86, 3.51)], indicating that this verb was used significantly more to describe the Reading behavior rather than for the Calibrating (*z* (297) = 8.97, *p* < 0.001) and Watching (*z* (297) = 9.09, *p* < 0.001) behaviors. We found a complementary main effect of the Behavior on the frequency of use of the verb “looking” [*β* = −1.99, *t* (293) = −3.24, *p* = 0.001, 95% *CI* = (−3.20, −0.79)], indicating that this latter verb was used less frequently to describe the Reading behavior than to describe the Calibrating (*z* (297) = −4.48, *p* < 0.001) or the Watching (*z* (297) = −3.84, *p* < 0.001) behaviors. We also found a trend of the Behavior on the frequency of use of the verb “watching” [*β* = 0.58, *t* (293) = 1.74, *p* = 0.082, 95% *CI* = (−0.07, 1.23)] that did not reach significance, but suggested that such verb was used to describe the Watching behavior more often than for the Reading behavior (*z* (297) = 4.33, *p* < 0.001). In addition, participants used the verb “watching” slightly more often after the Watching behavior than after the Calibrating one (*z* (297) = 2.16, *p* = 0.078), and more often after the Calibrating behavior than after the Reading behavior (*z* (297) = 2.63, *p* = 0.023).

**FIGURE 6:**
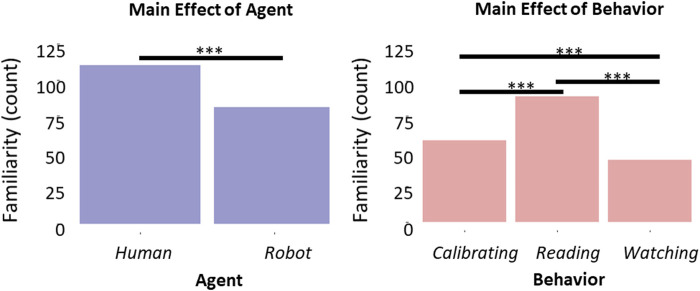
Histograms representing participants’ familiarity with the Agent **(A)** and with the Behavior **(B)**. Horizontal bars denote differences surviving post hoc comparison, asterisks define the level of significance of the comparison (*p < 0.05, **p < 0.01, *p* < 0.001).

When we analyzed the evaluation of familiarity attributed to the videos, we observed a main effect of both the Agent [*β* = −1.70, *t* (293) = −3.23, *p* = 0.001, 95% *CI* = (−2.72, −0.67); Figure 6A] and the Behavior [*β* = 2.74, *t* (293) = 3.15, *p* = 0.002, 95% *CI* = (1.04, 4.45); Figure 6B]. The main effect of the Agent indicated that videos depicting the human agent were rated as more familiar than videos depicting the iCub (*z* (297) = 3.94, *p* < 0.001). The main effect of the Behavior indicated that the Reading behavior was perceived as more familiar than both the Calibrating (*z* (297) = 4.82, *p* < 0.001) and Watching (*z* (297) = 6.10, *p* < 0.001) behaviors. Surprisingly, the Calibrating behavior was evaluated as slightly more familiar than the Watching behavior (*z* (297) = 2.38, *p* = 0.046).

Our analyses on participants’ ratings of anthropomorphic traits pointed out a systematic main effect of the Agent on all the attributes (i.e., “Naturalness,” “Awareness,” “Focus,” “Interest”). Specifically, the human always received higher ratings than the iCub (see Supplementary Materials for detailed comparisons). Furthermore, we found a systematic main effect of the Behavior, indicating that the Reading behavior received higher ratings than both the Calibrating and the Watching behaviors (see Supplementary Materials for detailed comparisons). There was no interaction effect between Agent and Behavior.

Finally, we compared whether the effects on participants’ ratings could be better explained by their Familiarity with the behaviors, rather than by the intrinsic characteristics of the Agent and the Behavior. For all comparisons, the most predictive models were the ones including the Agent and the Behavior as fixed factors, instead of the Familiarity (see Table 2 for detailed comparisons).

**TABLE 2:** Detailed Akaike’s Information Criterion (AIC) of models for each comparison.

Measure	Fixed Factor(s)	AIC	*χ2*	*P*
Naturalness	*Familiarity*	−1,040	–	
	Agent*Behavior	−1,085.5	53.52	<0.001
Awareness	*Familiarity*	−1,056.4	–	
	Agent*Behavior	−1,107.3	58.95	<0.001
Focus	*Familiarity*	−1,108.2	–	
	Agent*Behavior	−1,114	13.86	0.008
Interest	*Familiarity*	−959.93	–	
	Agent*Behavior	−984.17	32.248	<0.001

## Discussion—Experiment 2

With our second experiment, we tested individuals’ familiarity with the behaviors and agents used in Experiment 1. When asked to infer the Agents’ actions, participants were highly accurate in identifying the reading behavior, which we designed to be the “intentional” behavior of our stimuli. Indeed, the eye movements recorded during the watching and the calibrating behaviors were dependent on the occurrence of visual stimuli, or, in other words, to a bottom-up oculomotor capture (Troscianko and Hinde, 2011). On the other hand, the eye movements performed during the reading behavior were actively controlled by the agent himself, who was indeed displaying a top-down modulated action (Radach et al., 2008). Observing an “intentional” behavior (i.e., the reading behavior in our experiment) may elicit social cognitive mechanisms related to mindreading, which would, consequently, facilitate its identification. This facilitation may sound trivial when applied to a natural human-human interaction, as we usually assume human behavior to be driven by underpinning mental states and intentions (Dennett, 1971). However, it may be less intuitive when applied to artificial agents, as the same facilitation may not apply during observation of robot behavior. Indeed, robots do not possess a proper mind to read, but eliciting the ascription of a mind towards them could foster human-robot interaction, potentially smoothing the communication between natural and artificial systems (Wiese et al., 2017). Indeed, our result suggests that for our participants it was easier to identify the correct behavior when the action displayed was seemingly intentional. We claim that embedding intentional behaviors into embodied, artificial agents could boost social engagement by smoothing communication.

In line with this hypothesis, participants rated the reading behavior as more natural than the other behaviors. Furthermore, when either the human or the robot was displaying it, participants tended to rate the agent as more focused, interested, and aware. This suggests that behavioral cues of intentionality may affect individuals’ tendency to attribute anthropomorphic traits towards an artificial agent.

It is important to point out that participants perceived the reading behavior as the most familiar of the set, regardless of the agent that was displaying it. Additionally, the nature of the Agent affected the attribution of naturalness towards the Behavior, along with the perceived focus, interest, and awareness of the Agent (i.e., participants reported high familiarity with the videos that were depicting the human agent). However, the model comparisons revealed that the nature of the Agent and the Behavior explain our data better than the familiarity ratings alone. This supports the idea that familiarity alone cannot fully explain the differences we found in participants’ attributions. At the same time, we recognize that intentionality alone might not be the only factor affecting individuals’ attribution of anthropomorphic traits towards natural and artificial agents.

## General Discussion

In the current study, we presented two experiments aimed at investigating how individuals perceive and attribute human-likeness traits towards natural and artificial agents depending upon the level of “intentionality” displayed by their behaviors. Taken together, the results of both experiments suggest that observing a human and a humanoid displaying the same set of behaviors evokes different implicit attentional processes and, consequently, different explicit attributions.

Our first experiment highlighted the differences in spontaneous attentional engagement during the visual processing of the behavior displayed by the two agents. Processing behaviors that we designed to appear as “intentional” (i.e., controlled by the agent itself) required less attentional effort than “mechanistic” behaviors (i.e., purely stimulus-driven). Based on the results of our second experiment, we associate attentional engagement with the attribution of human-like traits towards the agent that displays the behavior. Indeed, in our second experiment, participants evaluated the seemingly intentional behavior as the most “anthropomorphic” of the set (i.e., agents displaying it seemed to be “more aware,” “focused,” “interested,” and “natural”). Additionally, the word-choice participant made to describe the behaviors was extremely accurate for the “intentional” ones, suggesting that it is easier for the observer to recognize the behavior of an artificial agent when the intent behind it is clear. It is important to point out that such facilitation does not depend solely upon the familiarity that participants perceived with the behaviors, but mostly on the degree of perceived intentionality and anthropomorphism. In other words, the degree of intentionality displayed by an artificial agent may affect attentional engagement, which, in turn, affects perceived familiarity and anthropomorphism. Thus, facilitating attentional engagement may be desirable to improve communication with artificial agents.

In this sense, endowing artificial agents with human-like behaviors, such as human-inspired eye movements, may boost communication and attunement towards them, a crucial aspect for deploying robots in environments where social interaction is inevitable (e.g., assistive robotics) (Leite et al., 2013). Our results bring further clarity to these hypotheses, highlighting the complex interplay between explicit attribution of anthropomorphic traits and attentional engagement. We claim that the attribution of anthropomorphic traits towards an artificial agent is the consequence of the perceived difficulty in processing the information related to its behavior. In turn, such perceived complexity may be modulated by the ease to ascribe intentions towards the artificial agent. However, it is important to point out that clarifying the causal relationship between attentional processing and attribution of anthropomorphism goes beyond the scope of the current work, and should be investigated in future research.

The acceptance of robots as social agents might depend upon their ability to elicit adequately the same social cognitive processes that are required during human-human interaction, even at an implicit level (Dennett, 1971). At the same time, their behavior needs to be easy to predict and to understand from the user perspective (Leite et al., 2013). In the last decade, we have been exposed to seemingly smart devices daily. Technological progress made the interaction with technology increasingly smooth and dynamic due to the implementation of human-like characteristics in the way artificial agents behave and communicate (González et al., 2012). The implementation of human-based and human-inspired behaviors in artificial agents may positively affect both implicit attentional processing and explicit attributions, and the spontaneity and naturalness of interaction (Dautenhahn, 2007). Furthermore, providing artificial agents with human-like behavior affects positively the quality of the interaction (Ghiglino et al., 2020b). In particular, when the physical aspect and the behavioral repertoire of artificial agents resemble one of the human beings, individuals tend to attribute spontaneously to the agent anthropomorphic traits, including mental states, intentional agency, and anthropomorphic traits (Ghiglino et al., 2020b). Subtle hints of human-likeness displayed by a humanoid robot seem to affect attentional engagement and attribution of anthropomorphic traits (see, for example, Martini et al., 2015; Thepsoonthorn et al., 2018). However, we demonstrated that such claims could not be generalized to all possible behaviors that artificial agents might display during spontaneous interaction with the users.

These promising results present new intriguing questions to be addressed in future studies. For instance, the choice of using a screen-based paradigm to study complex attentional mechanisms sacrifices some ecological validity of our results in favor of better experimental control. Indeed, when it comes to human-robot interaction and communication, embodiment plays a fundamental role (Kuniyoshi et al., 2004; Wainer et al., 2006; Deng et al., 2019; Ventre-Dominey et al., 2019). However, we found it necessary to test people’s sensitivity to subtle hints of human likeness using a systematic approach, first with in-lab experiments, and only later in naturalistic environments. Indeed, focusing on few isolated variables at a time, using well-controlled experimental designs, allows for minimizing the risk of confounding effects due to unforeseen variables. With this study, we demonstrated that implicit measures such as eye-tracking metrics can be adopted to study attentional mechanisms involved in the processing of artificial agents’ behaviors. We acknowledge that this approach was conservative, as it also led us to the investigation of a limited behavioral repertoire. In short, the study offers a reliable methodology that can be adapted to more interactive scenarios.

In conclusion, the current study supports the hypothesis that embedding robots with human-inspired behaviors may facilitate the interaction between them and humans. However, our results suggest that it is not sufficient to generate human-like behavior to ease the interaction. Besides, it may be crucial that the behavior exhibited by the agent displays traits that can be interpreted as intentional.

## Data Availability

The raw data supporting the conclusions of this article will be made available by the authors, without undue reservation.

## Footnotes

1 This is meant to facilitate communication in contexts where it is necessary to do so (i.e., healthcare application, such as motivating individuals to take medications), and not to deceive individuals about the nature of artificial agents.

2 Video comparison between the two agents displaying the two behaviors is available at: https://osf.io/b65ru/

3 Two agents, multiplied by two versions of each of the two behaviors, multiplied by two versions of each of the three backgrounds.

4 Factor 1: Agent (Human vs. Robot); Factor 2: Behavior (Reading vs. Calibrating vs. Watching).
