# Supplementary material for: Mind the Eyes: Artificial Agents’ Eye Movements Modulate Attentional Engagement and Anthropomorphic Attribution
Source: Front Robot AI. 2021 May 28;8:642796. doi: 10.3389/frobt.2021.642796 (PMC8192967; doi:10.3389/frobt.2021.642796)
Supplement: Supplementary file 1 [file DataSheet1.docx]

Supplementary Material

# Supplementary Tables

Table 3 – Average Fixation Duration (FD) divided per condition and AOI

| Agent | Behavior | Context | Average FD (Eye Region) | Average FD (Face Region) | Average FD (Background Region) |
| --- | --- | --- | --- | --- | --- |
| Human | Calibrating | Congruent | 101.25 | 69.04 | 68.08 |
|  |  | Incongruent | 98.91 | 78.65 | 66.26 |
|  |  | Neutral | 126.87 | 78.27 | 91.75 |
|  | Reading | Congruent | 98.07 | 61.36 | 95.31 |
|  |  | Incongruent | 99.16 | 70.39 | 93.68 |
|  |  | Neutral | 120.16 | 69.85 | 105.15 |
| Robot | Calibrating | Congruent | 89.46 | 70.54 | 62.85 |
|  |  | Incongruent | 85.76 | 63.74 | 63.96 |
|  |  | Neutral | 116.28 | 77.26 | 84.89 |
|  | Reading | Congruent | 88.44 | 75.71 | 76.36 |
|  |  | Incongruent | 92.33 | 73.45 | 75.53 |
|  |  | Neutral | 103.44 | 72.36 | 93.89 |

Table 4 – Detailed interactions and main effects on Fixation Duration (FD) in the AOI corresponding to eye region

| **Effect on FD** | t - Value | p value | β - Values | C.I. 2.5% | C. I. 97.5% |
| --- | --- | --- | --- | --- | --- |
| **Agent** | -2.21 | .028 | -11.795 | -22.141 | -1.449 |
| Behavior | -0.60 | .432 | -3.18 | -13.526 | 7.166 |
| Context | 3.52 | <.001 | 19.012 | 8.575 | 29.448 |
| Agent x Behavior | 0.29 | .746 | 2.16 | -12.472 | 16.791 |
| Agent x Context | 1.03 | .991 | 7.81 | -6.879 | 22.512 |
| Behavior x Context | 0.41 | .183 | 3.422 | -11.618 | 17.773 |
| Agent x Behavior x Context | -1.39 | .176 | -14.905 | -35.643 | 5.832 |

Table 5 – Average Fixation Proportion (FP) divided per condition and AOI

| Agent | Behavior | Context | Average FP (Eye Region) | Average FP (Face Region) | Average FP (Background Region) |
| --- | --- | --- | --- | --- | --- |
| Human | Calibrating | Congruent | 81.30 | 4.06 | 14.64 |
|  |  | Incongruent | 81.01 | 5.12 | 13.87 |
|  |  | Neutral | 79.83 | 5.71 | 14.46 |
|  | Reading | Congruent | 72.47 | 7.80 | 19.74 |
|  |  | Incongruent | 73.85 | 6.14 | 20.01 |
|  |  | Neutral | 74.16 | 6.50 | 19.34 |
| Robot | Calibrating | Congruent | 81.88 | 5.28 | 12.84 |
|  |  | Incongruent | 81.91 | 5.73 | 12.36 |
|  |  | Neutral | 82.46 | 6.02 | 11.52 |
|  | Reading | Congruent | 80.46 | 5.56 | 13.98 |
|  |  | Incongruent | 79.75 | 5.76 | 14.50 |
|  |  | Neutral | 79.71 | 6.43 | 13.86 |

Table 6 – Detailed interactions and main effects on Fixation Proportion (FP) in the AOI corresponding to eye region

| **Effect on FP** |  | t - Value | p value | β - Values | C.I. 2.5% | C. I. 97.5% |
| --- | --- | --- | --- | --- | --- | --- |
| Agent |  | 0.26 | .799 | 0.005 | -0.033 | 0.044 |
| Behavior |  | -5.85 | <.001 | -0.117 | -0.156 | -0.079 |
| Context |  | -1.29 | .891 | -0.026 | -0.065 | 0.013 |
| Agent x Behavior |  | 3.65 | <.001 | 0.103 | 0.049 | 0.158 |
| Agent x Context |  | 1.23 | .735 | 0.034 | -0.02 | 0.09 |
| Behavior x Context |  | 1.79 | .075 | 0.051 | -0.004 | 0.106 |
| Agent x Behavior x Context |  | -2.01 | .045 | -0.081 | -0.158 | -0.003 |

Table 7 – Average Decision Times (DTs) divided per condition

| Agent | Behavior | Context | Average DTs |
| --- | --- | --- | --- |
| Human | Calibrating | Congruent | 5372.65 |
|  |  | Incongruent | 5353.54 |
|  |  | Neutral | 5399.91 |
|  | Reading | Congruent | 4235.94 |
|  |  | Incongruent | 4204.50 |
|  |  | Neutral | 4316.05 |
| Robot | Calibrating | Congruent | 4502.33 |
|  |  | Incongruent | 4654.01 |
|  |  | Neutral | 4570.06 |
|  | Reading | Congruent | 4199.00 |
|  |  | Incongruent | 4181.08 |
|  |  | Neutral | 4167.55 |

Table 8 – Detailed interactions and main effects on Decision Times (DTs) in the AOI corresponding to eye region

| Effect on DTs | t - Value | p value | β - Values | C.I. 2.5% | C. I. 97.5% |
| --- | --- | --- | --- | --- | --- |
| Agent | -6.32 | <.001 | -0.181 | -0.237 | -0.126 |
| Behavior | -7.71 | <.001 | -0.221 | -0.276 | -0.165 |
| Context | 0.06 | .958 | 0.001 | -0.054 | 0.057 |
| Agent x Behavior | 4.25 | <.001 | 0.172 | 0.094 | 0.251 |
| Agent x Context | 0.23 | .604 | 0.028 | -0.068 | 0.088 |
| Behavior x Context | 0.35 | .679 | -0.015 | -0.064 | 0.092 |
| Agent x Behavior x Context | -0.67 | .800 | -0.038 | -0.149 | 0.072 |
